# Double maternal-effect: duplicated *nucleoplasmin 2* genes, *npm2a* and *npm2b,* with essential but distinct functions are shared by fish and tetrapods

**DOI:** 10.1186/s12862-018-1281-3

**Published:** 2018-11-12

**Authors:** Caroline T. Cheung, Jérémy Pasquier, Aurélien Bouleau, Thaovi Nguyen, Franck Chesnel, Yann Guiguen, Julien Bobe

**Affiliations:** 1 INRA LPGP UR1037, Campus de Beaulieu, 35042 Rennes, France; 2 CNRS/UMR6290, Université de Rennes 1, 35000 Rennes, France; 3 Laboratory of fish physiology and genomics (LPGP), National Institute of Agricultural Research (INRA), Campus de Beaulieu, 35042 Rennes Cedex, France

**Keywords:** *npm2*, Vertebrates, Evolution, Maternal-effect genes, CRISPR-cas9 knockout

## Abstract

**Background:**

*Nucleoplasmin 2* (*npm2*) is an essential maternal-effect gene that mediates early embryonic events through its function as a histone chaperone that remodels chromatin. Recently, two *npm2* (*npm2a* and *npm2b*) genes have been annotated in zebrafish. Thus, we examined the evolution of *npm2a* and *npm2b* in a variety of vertebrates, their potential phylogenetic relationships, and their biological functions using knockout models via the CRISPR/cas9 system.

**Results:**

We demonstrated that the two *npm2* duplicates exist in a wide range of vertebrates, including sharks, ray-finned fish, amphibians, and sauropsids, while *npm2a* was lost in coelacanth and mammals, as well as some specific teleost lineages. Using phylogeny and synteny analyses, we traced their origins to the early stages of vertebrate evolution. Our findings suggested that *npm2a* and *npm2b* resulted from an ancient local gene duplication, and their functions diverged although key protein domains were conserved. We then investigated their functions by examining their tissue distribution in a wide variety of species and found that they shared ovarian-specific expression, a key feature of maternal-effect genes. We also demonstrated that both *npm2a* and *npm2b* are maternally-inherited transcripts in vertebrates, and that they play essential, but distinct, roles in early embryogenesis using zebrafish knockout models. Both *npm2a* and *npm2b* function early during oogenesis and may play a role in cortical granule function that impact egg activation and fertilization, while *npm2b* is also involved in early embryogenesis.

**Conclusion:**

These novel findings will broaden our knowledge on the evolutionary history of maternal-effect genes and underlying mechanisms that contribute to vertebrate reproductive success. In addition, our results demonstrate the existence of a newly described maternal-effect gene, *npm2a*, that contributes to egg competence, an area that still requires further comprehension.

**Electronic supplementary material:**

The online version of this article (10.1186/s12862-018-1281-3) contains supplementary material, which is available to authorized users.

## Background

In animals and plants, early embryonic development relies strictly on maternal products until maternal-to-zygotic transition (MZT) during which zygotic genome activation (ZGA) occurs [1]. Maternal-effect genes are those that are transcribed from the maternal genome and whose products, which include transcripts, proteins, and other biomolecules, are deposited into the oocytes during their production in order to coordinate embryonic development before ZGA [2]. MZT is a key step that is needed firstly for clearance of maternal components, and secondly to activate zygotic gene expression and to allow subsequent embryonic development. Among the maternally-inherited transcripts that play important roles during early development, some were demonstrated to regulate zygotic program activation such as *nanog*, *pou5f1*, *soxb1* and microRNAs in zebrafish (*Danio rerio*) [3, 4]. Henceforth, all gene and protein nomenclature written in this manuscript will be based on that of zebrafish regardless of species for simplification purposes. Another gene, *nucleoplasmin 2* (*npm2*), belongs to the family of nucleoplasmins/nucleophosmins that was demonstrated in zebrafish to be maternally-inherited at both protein and mRNA levels, whereby both play important roles in early development [5]. Historically, this protein was identified and defined as a nuclear chaperone in *Xenopus* [6, 7]. While the protein has been shown to be the most abundant nuclear protein in the *Xenopus* oocyte [8] and to play a crucial role at fertilization due to its role in sperm chromatin decondensation [9, 10], its maternally-inherited mRNA has been recently demonstrated to be translated as a de novo synthesized protein that could play a crucial role during ZGA in zebrafish [5]. Further, *npm2* is one of the first identified maternal-effect genes in mouse whereby its deficiency results in developmental defects and eventual embryonic mortality [11].

The *npm2* gene belongs to the *npm* gene family that encompasses four members, *npm1, npm2, npm3*, *and npm4*. The evolution of the Npm family has been shown to result from the two rounds of whole genome duplication (WGD) that occurred in early vertebrates (vertebrate genome duplication 1 and 2, or VGD1 & VGD2, respectively) [12, 13]. Former evolutionary studies clearly provided a phylogenic model of this family; VGD1 produced two genes, *npm3/2* and *npm4/1*, from an ancestral *npm* gene and the following WGD, VGD2, further created the current four *npm* types with subsequent loss of *npm4* from mammals, but retained in most fish species [14–17]. Recently, two *npm2* genes were automatically annotated in the zebrafish genome, i.e. *npm2a* (ENSDARG00000076391) and *npm2b* (previously known as *npm2*, ENSDARG00000053963). As the teleost ancestor experienced an extra WGD event (TGD, or teleost-specific genome duplication) [18], doubling of genes and other types of genomic rearrangement may be present in teleost species compared with other vertebrates. Moreover, a fourth round of duplication occurred more recently in salmonids (salmonid-specific genome duplication or SaGD) [19, 20], leading to further possible doubling of genes and other genomic rearrangements. Multiple evolutionary events (impact of TGD, local duplication, etc.) could have led to the evolution of *npm2a*/*npm2b* in zebrafish, which in turn may have significant impact on vertebrate reproduction. Thus, we investigated the evolution, phylogenetic relationship, as well as biological functions of *npm2a* and *npm2b* in a wide range of vertebrate species to broaden our knowledge on the evolution of maternal-effect genes and the underlying mechanisms that contribute to reproductive success in vertebrates.

## Materials and methods

### Genomic databases

The following genomic data were extracted and investigated from the ENSEMBL genomic database (http://www.ensembl.org/index.html): human, *Homo sapiens*; mouse, *Mus musculus*; chicken, *Gallus gallus*; *Xenopus*, *Xenopus tropicalis*; coelacanth, *Latimeria chalumnae*; spotted gar, *Lepisosteus oculatus*; zebrafish, *Danio rerio*; and tetraodon, *Tetraodon nigroviridis*. The Chinese alligator (*Alligator sinensis*) genome was extracted and investigated from the NCBI genomic database (http://www.ncbi.nlm.nih.gov/genome/22419). The *Xenopus laevis* L and S genomes were analyzed from the Xenbase database (www.xenbase.org). Additional file 1 demonstrates all the species that were used in each analysis.

### Transcriptomic databases

Additional file 2 shows the databases that were used to extract the peptide sequences of all the investigated species. The following actinopterygian transcriptomes were retrieved and investigated from the Phylofish database (http://phylofish.sigenae.org/index.html): bowfin, *Amia calva*; spotted gar, *Lepisosteus oculatus*; elephantnose fish, *Gnathonemus petersi*; arowana, *Osteoglossum bicirrhosum*; butterfly fish, *Pantodon buchholzi*; European eel, *Anguilla anguilla*; rainbow trout, *Oncorhynchus mykiss*; allis shad, *Alosa alosa*; zebrafish, *Danio rerio*; panga, *Pangasius hypophthalmus*; northern pike, *Esox lucius*; grayling, *Thymallus thymallus*; Atlantic cod, *Gadhus morua*; medaka, *Oryzias latipes*; European perch, *Perca fluviatilis*; brown trout, *Salmo trutta*; European whitefish, *Coregonus lavaretus*; brook trout, *Salvelinus fontinalis*; Astyanax, *Astyanax mexicanus*; lake whitefish, *Coregonus clupeaformis*; eastern mudminnow, *Umbra pygmae*; and sweetfish, *Plecoglossus altivelis*.

### Gene predictions

#### Predictions of *npm2* genes

The peptidic sequences of zebrafish Npm2a and Npm2b were used as query in TBLASTN search to identify the open reading frame (ORF) encoding *npm2* genes in the various investigated genomes and transcriptomes. We also used sequences from human *npm2b* and *Xenopus npm2* genes to confirm the obtained sequences.

#### TBLASTN search

Genomic data were analyzed using the TBLASTN algorithm (search sensitivity: near exact match short) on the ENSEMBL website or the NCBI browser as indicated in Additional file 2. The TBLASTN algorithm on the SIGENAE platform was used on the transcriptomic data.

### Phylogenetic analysis

Amino acid sequences of 75 Npm2, 3 Npm3, and 3 Npm1 proteins were first aligned using ClustalW. The JTT (Jones, Taylor, and Thornton) protein substitution matrix of the resulting alignments was determined using ProTest software. Phylogenetic analysis of Npm proteins was performed using the Maximum Likelihood method (MEGA 5.1 software) with 1000 bootstrap replicates.

### Synteny analysis

Synteny maps of the conserved genomic regions in human, mouse, chicken, *Xenopus,* coelacanth, spotted gar, zebrafish, and tetraodon were produced using PhyloView on the Genomicus v75.01 website (http://www.genomicus.biologie.ens.fr/genomicus-75.01/cgi-bin/search.pl). Synteny analysis of the Chinese alligator conserved genomic regions was performed using TBLASTN searches in the corresponding genomic database. For each gene, the peptidic sequences of human and chicken were used as query, as far as they were referenced in the databases.

### RNA-seq

RNA-seq data were deposited into Sequence Read Archive (SRA) of NCBI under accession references SRP044781–84, SRP045138, SRP045098–103, and SRP045140–146. The construction of sequencing libraries, data capture and processing, sequence assembly, mapping, and interpretation of read counts were all performed as previously reported [21]. We datamined the Phylofish online database to retrieve the tissue localization data for *npm2a* and *npm2b.*

### Quantitative real-time PCR (qPCR)

For each sample, total RNA was extracted using Tri-Reagent (Molecular Research Center, Cincinnati, OH) according to the manufacturer’s instructions. Reverse transcription (RT) was performed using 1 μg of RNA from each sample as previously described [22]. Various tissues were harvested from wildtype (WT) zebrafish (*N* = 3), obtained from the INRA LPGP fish facility, and *Xenopus* (*N* = 3), obtained from the Institut de Génétique et Développement de Rennes, Université de Rennes 1 animal facility (Rennes, France). cDNA originating from the tissues of the individual fish was used as individual biological replicates while that from *Xenopus* was pooled and subsequently used for qPCR. Follicular oocytes at different stages of oogenesis were obtained from 3 to 4 different WT animals. The functional analyses were performed with individual clutches containing 50–200 embryos that were harvested and pooled from each *npm2a* (*N* = 4) and *npm2b* (*N* = 5) mutant female. qPCR experiments were performed on at least 3 clutches from each individual mutant, where each clutch represented a biological replicate, using the Fast-SYBR GREEN fluorophore kit (Applied Biosystems, Foster City, CA) with 200 nM of each primer in order to keep PCR efficiency between 90 and 100%, and an Applied Biosystems StepOne Plus instrument as per the manufacturer’s instructions. RT products, including control reactions, were diluted 1/25, and 4 μl of each sample were used for each PCR. All qPCR experiments were performed in triplicate. The relative abundance of target cDNA was calculated from a standard curve of serially diluted pooled cDNA and normalized to *18S*, *β-actin*, *β2-microglobulin*, and *ef1α* transcripts. The primer sequences can be found in Additional file 3.

### CRISPR-cas9 genetic knockout

Fish used in this study were reared and handled in strict accordance with French and European policies and guidelines of the INRA LPGP Institutional Animal Care and Use Committee, which approved this study. CRISPR/cas9 guide RNAs (gRNAs) were designed using the ZiFiT online software and were made against 3 targets within each gene to generate large genomic deletions, ranging from 250 to 1600 base pairs, that removed several exons which rendered the Npm2 proteins non-functional. Nucleotide sequences containing the gRNA were ordered, annealed together, and cloned into the DR274 plasmid. In vitro transcription of the gRNA from the T7 initiation site was performed using the Maxiscript T7 kit (Applied Biosystems), and their purity and integrity were assessed using the Agilent RNA 6000 Nano Assay kit and 2100 Bioanalyzer (Agilent Technologies, Santa Clara, CA). Zebrafish embryos at the one-cell stage were micro-injected with approximately 30–40 pg of each CRISPR/cas9 guide along with 8–9 nM of purified cas9 protein (a generous gift from Dr. Anne de Cian from the National Museum of Natural History in Paris, France). The embryos were allowed to grow to adulthood, and genotyped using fin clip and PCR that detected the deleted regions. The PCR products of the mutants were then sent for sequencing to verify the deletion. Once confirmed, the mutant females were mated with WT males to produce F1 embryos, whose phenotypes were subsequently recorded. Images were captured with a Nikon AZ100 microscope and DS-Ri1 camera (Tokyo, Japan).

### Genotyping by PCR

Fin clips were harvested from animals under anesthesia (0.1% phenoxyethanol) and lysed with 5% chelex containing 100 μg of proteinase K at 55 °C for 2 h and then 99 °C for 10 min. The extracted DNA was subjected to PCR using Advantage2 system (Clontech, Mountain View, CA) for *npm2b* and Jumpstart Taq polymerase (Sigma-Aldrich, St. Louis, MO) for *npm2a*. The primers are listed in Additional file 3.

### Histological analysis

Ovaries from age-matched WT (*N* = 3), and *npm2a*- (*N* = 3) and *npm2b*-mutant (*N* = 3) females were harvested, fixed in 4% paraformaldehyde, and embedded in paraffin. Then, at least 5 sections were cut at 7 μm thickness from 5 different areas of the ovary from each animal, and the slides were stained with Regaud’s hematoxylin. Images were captured using a Nikon Eclipse microscope and camera set. The number and size of the CGs as well as total area of all the stage II follicles (according to Kimmel et al. [23] and Selman et al. [24]) in all the slides were quantitated using ImageJ software (National Institute of Health).

### Statistical analysis

Comparison of two groups was performed using the GraphPad Prism statistical software (La Jolla, CA), and either the Student’s t-test or Mann-Whitney U-test was conducted depending on the normality of the groups based on the Anderson-Darling test.

## Results and discussion

### Evolution of *npm2a* and *npm2b* in vertebrates

As previously shown, *npm1*, *npm2*, *npm3* and *npm4* genes are thought to have originated from the first two rounds of whole genome duplication (WGD) in the ancestral vertebrate (VGD1 and VGD2), which occurred early on in vertebrate evolution [14–17]. However, two *npm2* genes have been automatically annotated in the zebrafish genome, i.e. *npm2a* and *npm2b*.We set out to investigate the evolutionary history and function of the newly annotated *npm2a* gene and compare it with its homolog, *npm2b*. In order to verify if these two *npm2* genes are paralogous to each other and to determine their origin, we used a Blast search approach in various public databases to retrieve 91 sequences that could be related to *npm2* genes. We used sequences from evolutionarily diverse species such as zebrafish and *Xenopus npm2* genes as well as human *npm2b* to ascertain that all paralogs from more distantly related species were obtained. All retrieved sequences are compiled in Additional file 2.

### Phylogenetic analysis

**Fig. 1:**
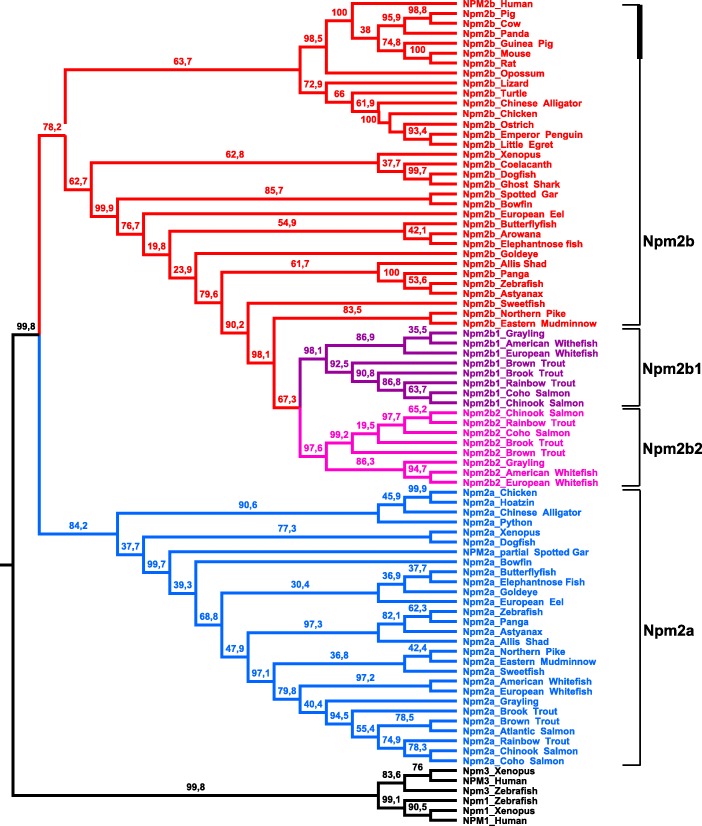
Consensus phylogenetic tree of nucleoplasmin 2 (Npm2) proteins. This phylogenetic tree was constructed based on the amino acid sequences of Npm2 proteins (for the references of each sequence see Additional file 2) using the Maximum Likelihood method with 1000 bootstrap replicates. The number shown at each branch node indicates the bootstrap value (%). The tree was rooted using Npm3 and Npm1 sequences. The Npm2a sequences are in blue, the Npm2b sequences are in red, the salmonid Npm2b1 sequences are in purple, and the salmonid Npm2b2 sequences are in pink

In order to verify that the retrieved protein sequences (Additional file 2) are homologous to zebrafish Npm2a and Npm2b, a phylogenetic analysis on Npm2 was performed. Based on the alignment of 77 vertebrate Npm2-related sequences, and using vertebrate Npm1 and Npm3 amino acid sequences as out-groups, a phylogenetic tree was generated (Fig. 1). We found that species from various vertebrate groups, including chondrichthyans (such as dogfish), ray-finned fish, lobe-finned fish, amphibians, as well as sauropsids contained both Npm2a and Npm2b sequences. However, no Npm2a sequence was identified in mammals, including human, pig, cow, panda, guinea pig, mouse, rat, and opossum although they all harbor Npm2b. These results demonstrated, for the first time, that proteins previously reported as Npm2 (*Xenopus*, cattle, mouse, human, zebrafish) are orthologous to Npm2b in all investigated vertebrate species, and should therefore now be referred to as Npm2b. Further, neither Npm2a nor Npm2b sequences were found in neoteleosteans including medaka, European perch, Atlantic cod, and tetraodon as well as in lampreys. In contrast, our analysis demonstrated the existence of two paralogous Npm2b proteins (i.e. Npm2b1 and Npm2b2) in all of the investigated salmonid species (rainbow trout, brown trout, and brook trout).

The existence of the two Npm2 clades indicated that Npm2a and Npm2b are likely paralogous to each other. In addition, comparison of Npm2a and Npm2b amino acid sequences in the species that harbor both revealed that they share between 30.2% and 46.4% homology, depending on the species (Additional file 4). The low sequence identity is consistent with an ancient duplication event that gave rise to *npm2a* and *npm2*b genes. However, neither the topology of the Npm2 phylogenetic tree nor the comparison between Npm2a and Npm2b could indicate the kind of duplication event that had occurred. In contrast, the high sequence identity shared by Npm2b1 and Npm2b2 in salmonids (between 75.1% and 86%) suggested a more recent duplication event. This observation in all of the investigated salmonid species is consistent with the hypothesis that *npm2b1* and *npm2b2* genes likely resulted from the SaGD.

### Synteny analysis

**Fig. 2:**
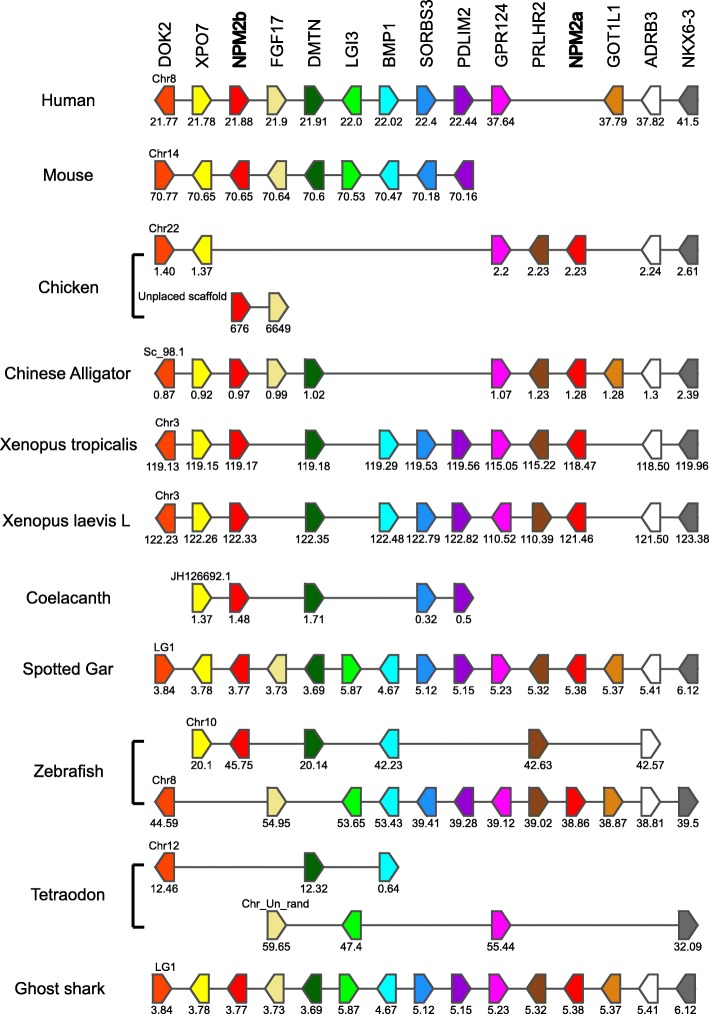
Conserved genomic synteny of *nucleoplasmin 2*, *npm2*, genes. Genomic synteny maps comparing the orthologs of *npm2a, npm2b*, and their neighboring genes. *npm2* genes are named as *npm2a* and *npm2b* (formerly known as *npm2*). The other genes were named after their human orthologs according to the Human Genome Naming Consortium (HGNC). Orthologs of each gene are shown in the same color. The direction of arrows indicates the gene orientation, with the ID of the genomic segment indicated above and the position of the gene (in 10^− 6^ base pairs) indicated below. The full gene names and detailed genomic locations are given in Additional file 5

In order to further understand the origin of the *npm2a* and *npm2b* genes in vertebrates, we performed a synteny analysis of their neighboring genes in representative vertebrate genomes. We focused our study on two mammals (human and mouse), two sauropsids (Chinese alligator and chicken), two amphibians (*Xenopus tropicalis* and *laevi*s L), one basal sarcopterygian (coelacanth), one basal actinopterygian (spotted gar), and two teleosts (zebrafish and tetraodon) (Fig. 2). Analysis was also performed on the *Xenopus laevis* S subgenome, but since the results were very similar to that of *Xenopus laevis* L subgenome, we only showed the latter’s data [25].

The human, mouse, Chinese alligator, *Xenopus tropicalis* and *laevis*, coelacanth, spotted gar, and zebrafish *npm2b* genes are located in genomic regions containing common loci, including *dok2, xpo7, fgf17, dmtn, lgi3, bmp1, sorbs3, pdlim2, gpr124, prlhr, got1l1, adrb3,* and *nkx6–3*. Together with the phylogenetic analysis, this indicated that *npm2b* genes investigated here are orthologous. Synteny analysis confirmed the absence of the *npm2b* gene in tetraodon although the above-mentioned neighboring genes are present in its genome (Fig. 2 and Additional file 5).

The Chinese alligator, chicken, *Xenopus tropicalis* and *laevis*, spotted gar, and zebrafish *npm2a* genes are located in genomic regions containing the same loci as *npm2b* conserved regions (Fig. 2). Indeed, Chinese alligator and spotted gar *npm2a* and *npm2b* genes are located in the vicinity of each other on scaffold 98.1 and the linkage group LG1, respectively. The presence of *npm2a* and *npm2b* genes in the same genomic region in representative species of sarcopterygians (Chinese alligator and *Xenopus tropicalis* and *laevis*) and actinopterygians (spotted gar) strongly suggested that *npm2a* and *npm2b* genes could have resulted from a unique local duplication of an ancestral *npm2* gene.

### Evolutionary history of npm2 genes in vertebrates

**Fig. 3:**
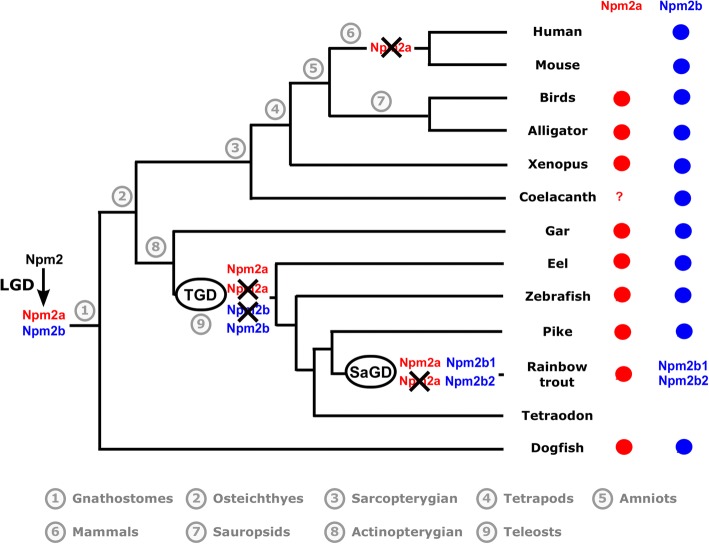
Current status and proposed evolutionary history of *nucleoplasmin 2* (*npm2*) genes among gnathostomes. The names of the current representative species of each phylum are given at the end of the final branches, together with a red and/or blue X to denote the *npm2* genes they possess (*npm2a* = red, *npm2b* = blue). The black X upon an *npm2* gene symbol indicates a gene loss. LGD: local gene duplication; TGD: teleost-specific whole genome duplication; SaGD: salmonid-specific whole genome duplication

The presence/absence of *npm2a* and *npm2b* in the current vertebrate phyla and species is summarized in Fig. 3, and we also propose an evolutionary scenario for the diversification of the *npm2* genes across vertebrate evolution.

In this study, we demonstrated that *npm2a* and *npm2b* may be paralogous genes present in the different vertebrate groups, chondrichthyans (Fig. 1), sarcopterygians, and actinopterygians (Figs. 1 and 2), which strongly suggested that the *npm2* genes originated from a duplication event prior to the divergence of chondrichthyans and osteichthyans. Since the four *npm* family members (*npm1*, *npm2*, *npm3*, and *npm4*) are thought to be produced from the first two rounds of WGD (VGD1 & VGD2) that occurred early on in vertebrate evolution, we can thus hypothesize that the duplication event that generated *npm2a* and *npm2b* took place after VGD2, but before emergence of chondrichthyans and osteichthyans, between 450 and 500 million years ago (Mya) [26]. The findings from our synteny analysis demonstrated that in representative species of actinopterygians (spotted gar) and sarcopterygians (*Xenopus tropicalis* and *laevis* and Chinese alligator), *npm2a* and *npm2b* genes are at two distinct loci located on the same chromosomic region (Fig. 2). These results strongly suggested that *npm2a* and *npm2b* genes arose from local gene duplication rather than a whole genome (or chromosome) duplication event (Fig. 3).

The teleost ancestor experienced an extra WGD event (TGD) [18], but in all investigated teleosts, we observed a maximum of one *npm2a* ortholog and one *npm2b* ortholog (except in salmonids) (Fig. 1). In addition, *npm2a* and *npm2b* are located on two TGD ohnologous regions on chromosomes 8 and 10, respectively (Fig. 2), in zebrafish, which is consistent with the loss of one of the *npm2a* duplicates from one region and the loss of one *npm2b* duplicate from the corresponding ohnologous region. Thus, *npm2* evolution is most likely due to the early loss of one of the two *npm2a* and *npm2b* TGD ohnologs. This observation in zebrafish strengthened the hypothesis that TGD did not impact the *npm2a* and *npm2b* evolution in teleosts. In addition, the lack of *npm2a* and *npm2b* in neoteleostei species, such as tetraodon, suggested that additional gene losses occurred early in the evolutionary history of this group (Fig. 2).

In salmonids, we identified two *npm2b* paralogs, i.e. *npm2b1* and *npm2b2*, in all investigated salmonid species (Fig. 1). Considering the high amino acid sequence identity shared by Npm2b1 and Npm2b2 (between 75.1% and 86%), it is strongly hypothesized that these duplicates originated from SaGD. In contrast, we identified only one *npm2a* gene in all investigated salmonids suggesting that SaGD did not impact the current salmonid *npm2a* evolution most likely due to early loss of the SaGD ohnolog of this gene (Fig. 3).

In addition to the early gene losses after WGD, various other independent and phylum-specific gene losses may have contributed to shape the current *npm2a* and *npm2b* genes in vertebrates. In fact, although both *npm2a* and *npm2b* have been globally conserved in sarcopterygians and actinopterygians, some phyla in each group lack at least one of the genes. In sarcopterygians, *npm2a* is conserved in amphibians such as *Xenopus tropicalis* and *laevis*, and in sauropsids such as Chinese alligator and chicken (Figs. 1, 2, 3). In contrast, we did not find any *npm2a* genes in the Coelacanth genome (Figs. 1, 2, 3), which could be due to the lower assembly quality of the concerned genomic region (see Additional file 5), and in the mammalian genomes including human and mouse (Figs. 1, 2, 3), whose absence may have been due to the loss of this gene in the common ancestor of mammals. Further, neither gene was found in lampreys which suggests early loss of the ancestral gene in this group. Thus, our data suggest that *nmp2a* was retained in vertebrates with late (i.e. mid-blastula) ZGA such as fish and amphibians, while it was lost in mammals that have early (i.e. 1–3 embryonic divisions) ZGA [27]. Considering the essentialness of the *npm2* gene in embryonic development, its lack in some neoteleostean species, such as Atlantic cod and medaka, raises the question on how evolution can cope with its loss. Further analyses of data from the Phylofish database [21] (data not shown) showed that *npm3* had the strongest homology to *npm2* and was predominantly expressed in the ovaries in medaka and cod which suggest that it could potentially compensate for *npm2* deficiency. In contrast, *npm3* does not show an ovarian-predominant expression in spotted gar and zebrafish, thus suggesting that its strong ovarian expression could be restricted to neoteleostei.

### Peptidic domains of npm2 paralogs

To gain insight into the functional role of Npm2a, we examined its protein domains in comparison with Npm2b since to date, only the role of Npm2b has been investigated, such as in *Xenopus tropicalis* [9, 28, 29], mouse [11, 30], humans [31–33], cattle [34], and zebrafish [5]. However, no data is available on the structure and function of Npm2a. The nucleoplasmin family is defined by the presence of an Npm core domain, which enables oligomerization of Npm proteins, in all of its members [35, 36]. We were able to predict the presence of this domain in all investigated Npm2a and Npm2b sequences (Additional file 6), suggesting that Npm2a proteins could also form homo- or hetero-polymers. Acidic tract domains are also features of nucleoplasmin proteins and were demonstrated to facilitate histone binding by increasing the recognition and affinity for different histones [37, 38]. Acidic tract A1 was demonstrated to be absent from most of the Npm2b investigated so far, except *Xenopus tropicalis* Npm2b, and acidic tract A2 was predicted to be in all investigated Npm2b apart from guinea pig Npm2b and American whitefish Npm2b1 (Additional file 6). This strongly suggested that the histone and basic protein binding activity of Npm2b is mediated predominantly by acidic tract A2. On the other hand, in all investigated Npm2a proteins, we predicted an acidic tract A1 except in that of dogfish, zebrafish, and allis shad (Additional file 6). In contrast, only half of the investigated Npm2a proteins harbored an acidic tract A2, which is additionally shorter than the one present in Npm2b proteins (Additional file 6). Our results demonstrated that most investigated Npm2a proteins possess acidic tracts that could potentially mediate histone and basic protein interactions, and that are different than those found in Npm2b which suggest that these two proteins may have diverse functions.

As previously demonstrated, *npm2b* functions as a histone chaperone to decondense sperm DNA as well as reorganize chromatin, thus, it has also been suggested to contribute to ZGA as well. [5, 11, 34, 39] Npm2b is thought to be activated by various post-translational modifications and homo-pentamerisation [40], and subsequently interacts with chromatin by exchanging sperm-specific basic proteins with histones via its core domain and acidic tracts A1, A2 and A3 [32, 36, 38, 41–44]. We found that most of the investigated Npm2a proteins possess acidic tract A1 while some have a shortened A2, in contrast to Npm2b proteins which have a long A2 and mostly lack A1. Thus, Npm2a is likely capable of mediating histone and basic protein interactions like the other nucleoplasmins, although its function may be different than that of Npm2b.

### Expression profiles of *npm2a* and *npm2b*

**Fig. 4:**
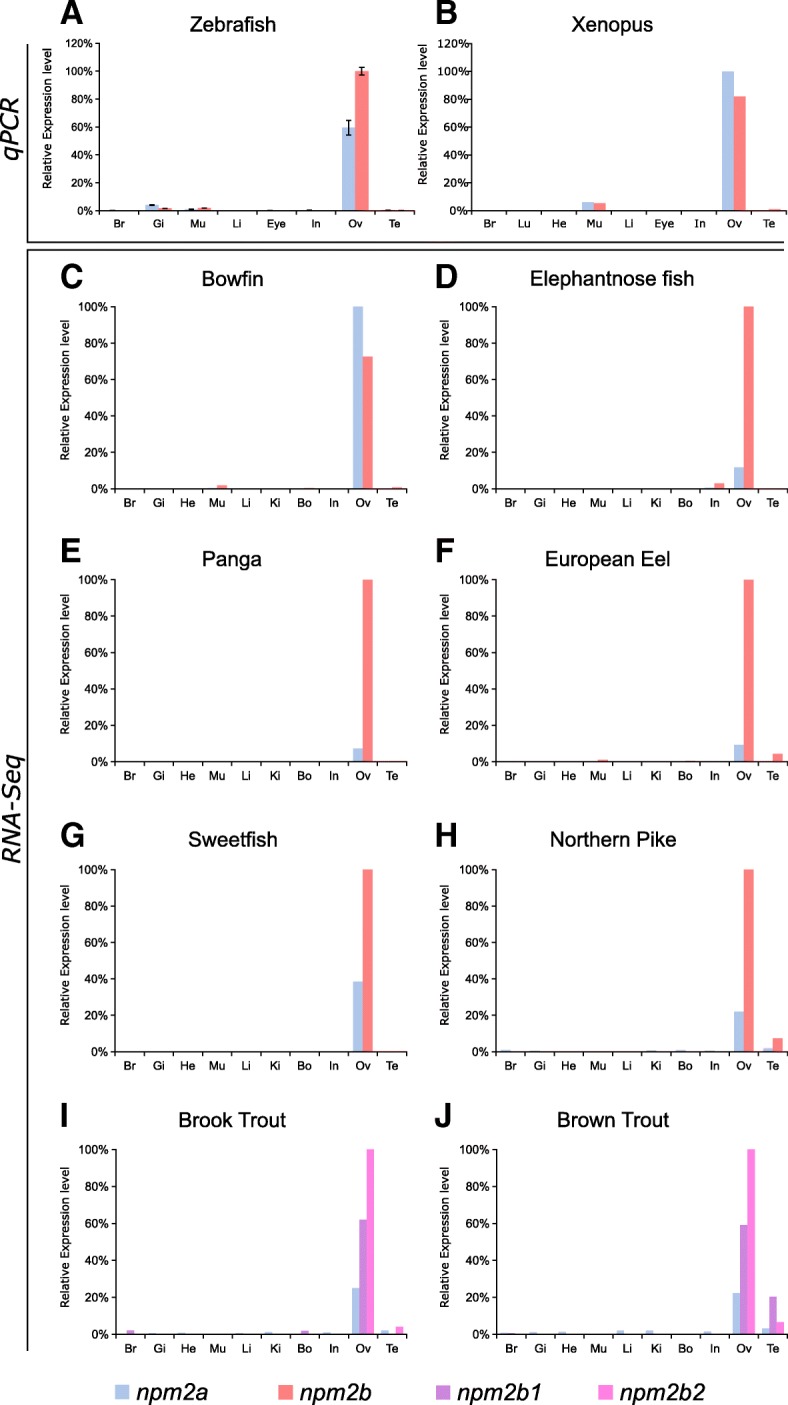
Tissue distribution of *nucleoplasmin* (*npm*) *2a* and *npm2b* in different species. Tissue expression analysis by quantitative real-time PCR of *npm2a* and *npm2b* mRNAs in (**a**) zebrafish and (**b**) *Xenopus*. Expression level is expressed as a percentage of the expression in the ovary for the most expressed gene. Data were normalized using *18S* expression. *N* = 3 individual animals. (**c**-**j**) Tissue expression level by RNA-Seq of *npm2a* and *npm2b* mRNAs in different fish species. mRNA levels are expressed in read per kilobase per million reads (RPKM). In salmonids (**i** and **j**), the two ohnologs of *npm2b* are *npm2b1* and *npm2b2*. Br, brain; Gi, gills; Lu, lung; In, intestine; Li, liver; Mu, muscle; He, heart; Bo, bone; Ki, kidney; Ov, ovary; and Te, testis

In order to investigate further the potential functions of Npm2a and Npm2b, we explored the tissue distributions of both transcripts using two different approaches, qPCR and RNA-seq, the latter of which was obtained from the Phylofish online database [21]. In zebrafish and *Xenopus tropicalis*, we observed by qPCR that *npm2a* and *npm2b* were both predominantly expressed in the ovary, and to a lesser extent, the muscle, as well as in the zebrafish gills (Fig. 4a and b). We also demonstrated via the Phylofish database that *npm2a* and *npm2b* were predominantly expressed in the ovary of bowfin, elephantnose fish, panga, European eel, sweetfish, and northern pike (Fig. 4c to h). In the investigated salmonid species, brook trout and brown trout, *npm2a*, *npm2b1*, and *npm2b2* were also predominantly expressed in the ovary (Fig. 4i and j). In addition, *npm2* transcripts were expressed at a very low level in the testis of European eel, northern pike, and salmonid species (Fig. 4f, h, i, and j). In teleosts, *npm2a* mRNA levels globally tended to be lower than *npm2b* (or *npm2b1* and *npm2b2* for salmonids) (Fig. 4a and d-j).

**Fig. 5:**
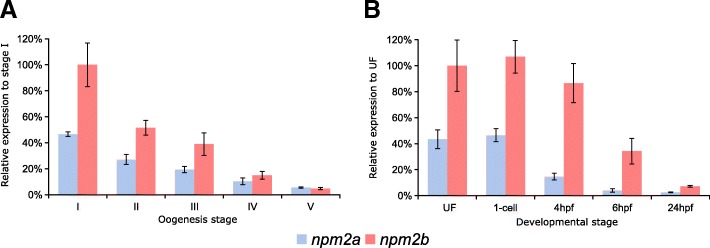
*Nucleoplasmin* (*npm*) *2a* and *npm2b* expression during oogenesis and early development. Quantitative real-time PCR analysis of *npm2a* and *npm2b* expression during (**a**) oogenesis and (**b**) early embryonic development in zebrafish. Data were normalized using luciferase, and relative expression was based on *npm2b* expression at the indicated stages (stage I during oogenesis and UF during embryogenesis). UF, unfertilized egg; hpf, hours post-fertilization. *N* = 3–4 individual animals

In order to delve deeper into their functions in reproduction and embryogenesis, we investigated *npm2a* and *npm2b* mRNA expression during oogenesis and early development in zebrafish. During zebrafish oogenesis, both *npm2a* and *npm2b* transcripts were found at high levels in oocytes (Fig. 5a), and despite gradual decreases in the levels of both *npm2* during oogenesis, they still can be detected at reasonable amounts in the unfertilized egg (i.e. metaphase 2 oocyte) (Fig. 5b). Thereafter, *npm2a* and *npm2b* transcript levels progressively decreased during embryonic development after fertilization with a substantial reduction after MBT (around 4 hpf) before reaching very low levels at 24 h post-fertilization (hpf) (Fig. 5b).

Our findings demonstrated clear ovarian-specific expression profiles of *npm2a* and *npm2b* transcripts that were conserved in all investigated species, from teleosts to tetrapods, which suggested that they both have roles in female reproduction and/or embryonic development. This is consistent with the *npm2b* mRNA profiles reported in the literature for mouse [11], cattle [34], *Xenopus tropicalis* [39], and zebrafish [5]. Further, we found that the *npm2* transcripts were highly abundant from early oogenesis through to early embryogenesis, finally being degraded at around MBT, which suggested that both mRNAs are strictly maternal (i.e. not re-expressed by the zygote), consistent with previous studies on zebrafish *npm2a* and *npm2b* transcripts [5, 45]. Their expression profiles are typical features of maternally-inherited mRNAs, which highly suggested that the newly described *npm2a* is also a maternal-effect gene as well.

### Functional analysis of npm2a and npm2b in zebrafish

**Fig. 6:**
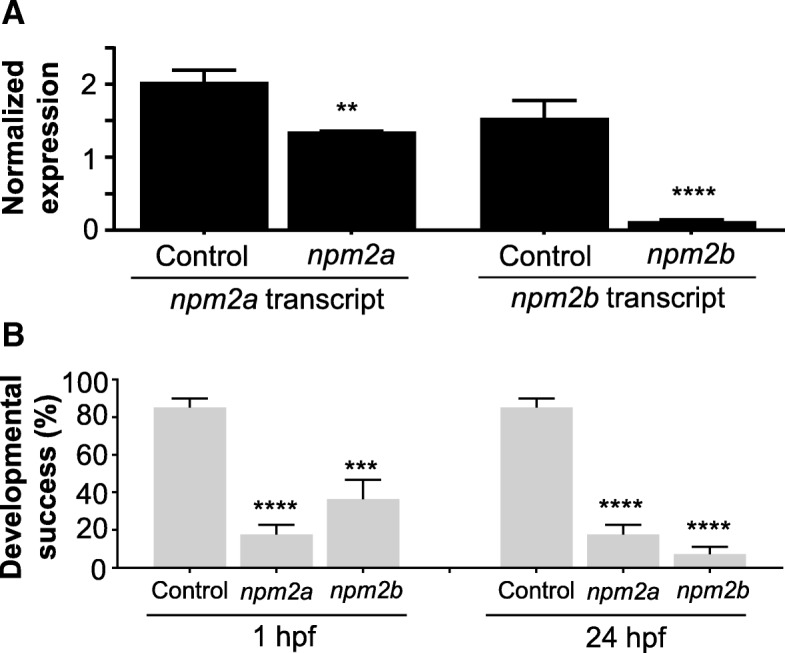
CRISPR/cas9 knockout of *nucleoplasmin* (*npm*) *2a* and *npm2b* in zebrafish. (**a**) Normalized expression level of *npm2a* and *npm2b* transcripts by quantitative real-time PCR (qPCR) in the fertilized zebrafish eggs from crosses between *npm2a* mutant, *npm2b* mutant, or wildtype (WT) (control) female and WT male, respectively. (**b**) Developmental success as measured by the proportion of fertilized eggs that underwent normal cell division and reached normal developmental milestones based on Kimmel et al. [23] from crosses between WT animals (control), and *npm2a* or *npm2b* mutant female and WT male at 1 and 24 h post-fertilization (hpf). qPCR data were normalized to *18S*, *β-actin*, *β2-microglobulin*, and *ef1α*. *N* = 4 each for *npm2a* mutant and control, and *N* = 5 for *npm2b* mutant. All assessments were performed from at least 3 clutches from each mutant. **p < 0.01, ***p < 0.001, ****p < 0.0001 by Mann-Whitney U-test

To understand the roles of these two *npm2* proteins during oogenesis and early embryogenesis, we performed functional analysis of these two Npm2 proteins by genetic knockout using the CRISPR/cas9 system. One-cell staged embryos were injected with the CRISPR/cas9 guides that targeted either *npm2a* or *npm2b* and allowed to grow to adulthood. Since the mutagenesis efficiency of the CRISPR/cas9 system was very high, as previously described [46, 47], the *npm2* genes were sufficiently knocked-out even in the mutant mosaic F0 females. This was evidenced by the substantially lower transcript levels of *npm2a* and *npm2b* in the F1 embryos as compared to those from control WT pairings (Fig. 6a). Thus, the phenotypes of *npm2a* (*n* = 4) and *npm2b* (*n* = 5) mutants could be observed even in the F0 generation. Since none of the mutated genes were transmissible to future generations neither through the male nor the female (ie. all the surviving embryos were WT), therefore, all of our observations were obtained from the F0 generation.

**Fig. 7:**
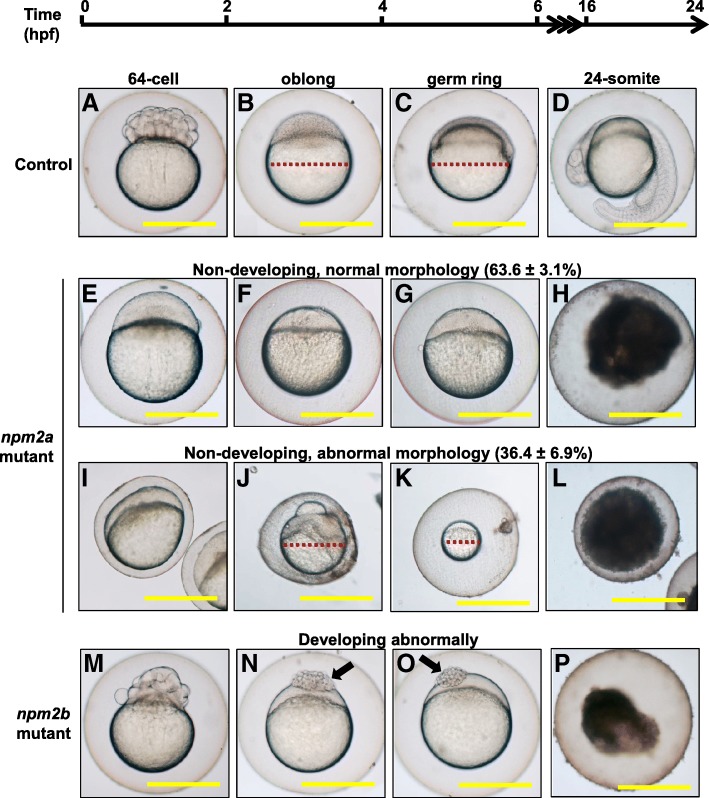
Effect of *nucleoplasmin* (*npm*) *2a* and *npm2b* deficiencies on zebrafish embryogenesis. Representative images demonstrating development of fertilized eggs from crosses between control (**a**-**d**), *npm2a* (**e**-**l**), or *npm2b* (**m**-**p**) females and wildtype (WT) males from 2 to 24 h post-fertilization (hpf). In the control eggs, the embryos were at 64-cell (**a**), oblong (**b**), germ ring (**c**), and 24-somite (**d**) stages according to Kimmel et al. [23]. Eggs from *npm2a* mutant females were non-developing with a normal morphology (**e**-**h**) or with an abnormal morphology (**i**-**l**). Eggs from *npm2b* mutant females had a normal morphology albeit were developing abnormally (**e**-**h**). (**a**, **e**, **i**, **m**) = images taken at 2 hpf; (**b**, **f**, **j**, **n**) = images taken at 4 hpf; (**c**, **g**, **k**, **o**) = images taken at 6 hpf; (**d**, **h**, **l**, **p**) = images taken at 24 hpf. Scale bars denote 400 μm. Red dotted lines define the diameter of the embryo. Arrows demonstrate a partially cellularized blastodisc that was sitting atop an enlarged yolk syncytial layer

We observed that most of the embryos from both *npm2a* and *npm2b* mutant females had a very low developmental success even at a very early stage of growth (1 hpf) (17.6 ± 5.1% and 36.4 ± 10.2%, respectively, vs. 85.1 ± 4.9% in controls) (Fig. 6b) as defined by a complete lack of cell division (non-cellularized). Table 1 demonstrates the penetrance of the *npm2a* and *npm2b* mutant phenotypes in single spawns from individual mutants. Relative to the controls (Fig. 7a-d), the *npm2a* mutant females produced non-developing eggs with two phenotypes; those with normal morphology (Fig. 7e-h), and a large population with an abnormal morphology (Fig. 7i-l), which included smaller egg, enlarged yolk, and not fully expanded chorion membrane (36.4 ± 6.9% vs. 0% in controls). The diameter of the egg is demonstrated by the red dotted lines, which reveal the extremely reduced size of some of the *npm2a* embryos (Fig. 7b, c, j, k). The eggs derived from *npm2a* mutant females that did not undergo any cell division at 1 hpf continued to display a complete lack of development up to 8 hpf. By 24 hpf, the non-developing eggs of both phenotypes from *npm2a* mutant females that failed to divide were all dead while the remaining embryos that showed normal development and cell division continued to progress normally. The observed phenotype of the non-cellularized *npm2a* embryos was very similar to previously described unfertilized eggs [48]. These novel findings showed for the first time that *npm2a* is essential for the developmental competence of zebrafish eggs, and is therefore a crucial maternal-effect gene.

**Table 1:** Defects in F1 embryos derived from crossing wild type males to F0 females that had been injected with *npm2a* or *npm2b* CRISPR mRNA as embryos

Penetrance of *npm2a* and *npm2b* mutant phenotypes					
	Total number of embryos	Embryos with defects			
		Non-cellularized^†^	Partially cellularized^‡^	Normal embryos	% Abnormal embryos
*npm2a-1*	741	366	0	375	49%
*npm2a-2*	252	237	0	15	94%
*npm2a-3*	107	82	0	25	77%
*npm2a-4*	150	147	0	3	98%
*npm2b-1*	105	47	42	16	85%
*npm2b-2*	66	60	6	0	100%
*npm2b-3*	263	129	134	0	100%
*npm2b-4*	525	347	173	5	99%
*npm2b-5*	376	100	92	184	51%

*npm2a* nucleoplasmin 2a, *npm2b* nucleoplasmin 2b

This table demonstrates representative data from a single clutch from each mutant female. ^†^Embryos did not develop at all (please refer to Fig. 7e-l). ^‡^Embryos had a partially cellularized blastodisc that was sitting atop an enlarged yolk syncytial layer (please refer to arrows in Fig. 7n and o)

In addition to the eggs that never developed past the one-cell stage, a significant number of the *npm2b*-derived eggs (29.2 ± 7.3% vs. 0% in controls) showed abnormal cell division that culminated in developmental arrest at around 4 hpf or the MBT following which the cells stopped dividing and appeared to regress (Fig. 7m-p). These eggs had a partially cellularized blastodisc that was sitting atop an enlarged yolk syncytial layer that became larger with time (arrows in Fig. 7n and o). By 24 hpf, these embryos were all dead while the remaining embryos that showed normal development and cell division continued to progress normally (7.2 ± 3.9% vs. 85.1 ± 4.9% in controls).

Our findings demonstrated that *npm2a* deficiency leads to a phenotype of decreased egg competence at the earliest stage whereby most of the spawned eggs failed to undergo cell division and subsequently died. Eggs that were deficient in *npm2b* also showed lack of development beyond the one-cell stage, but a proportion of these eggs were able to overcome this deficiency possibly due to residual expression of *npm2b* or compensation by other molecules, and these eggs were fertilized and underwent several cell divisions before arresting and eventually dying. This latter phenotype corroborated with that described in a previous study which demonstrated that fertilized eggs injected with morpholinos against *npm2b* were arrested at around MBT and eventually died [5]. Our novel results therefore suggested that *npm2a* functions predominantly during early oogenesis; its deficiency is completely incompatible with development and cannot be compensated by other molecules. On the other hand, *npm2b* plays a nonessential role during early oogenesis and early development, and its primary function is likely during MBT as previously described.

**Fig. 8:**
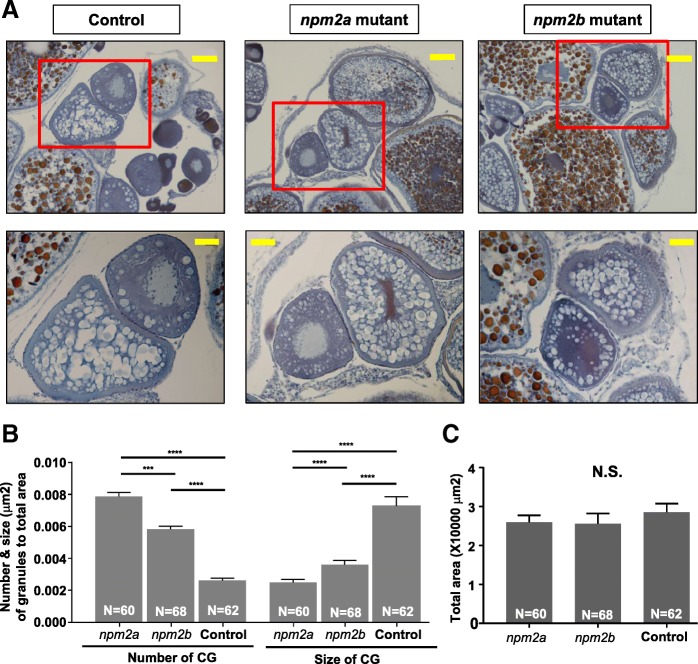
Histological analyses of the ovaries from *nucleoplasmin* (*npm*) *2a* and *npm2b* mutant animals. **a** Ovary sections from age-matched *npm2a*, *npm2b*, and wildtype (WT) control females stained with Regaud’s hematoxylin. Top panels, 20X magnification; bars denote 90 μm; bottom panels show higher magnification images of the boxed areas in the top panels, 40X magnification; bars denote 45 μm. **b** Quantitation of the number and size (μm) of the cortical granules after adjusting to the total area of stage II follicles from ovaries from age-matched *npm2a*, *npm2b*, and control females. The N number denotes the total number of follicles counted for each group. **c** Total area of the stage II follicles from ovaries from age-matched *npm2a*, *npm2b*, and control females. *N* = 3 for all samples. N.S. = not significant, ***p < 0.001, ****p < 0.0001 by Mann-Whitney U-test

In order to further characterize the phenotypes of these mutants, histological analyses of their ovaries were performed. We found that there were distinct differences in the appearance of stage II follicles (140–340 μm in diameter) [23, 24]. In particular, there was a drastic increase in number and reduction in size of the cortical alveoli or granules (CGs) in the stage II follicles of both *npm2* mutants compared with those in the wildtype controls (Fig. 8a). There was a substantial progressive increase in number of CGs from wildtype controls (0.0026 ± 0.0002 CGs/μm^2^; *p*-value< 0.0001, *npm2a* and *npm2b*) to *npm2b* mutant follicles (0.0065 ± 0.0002 CGs/μm^2^; *p*-value = 0.0002, *npm2a*) to *npm2a* mutant follicles (0.0079 ± 0.0003 CGs/μm^2^) after adjusting to the total area of the follicle that was investigated (Fig. 8b). There was also a progressive decrease in the size of CGs from wildtype controls 0.0073 ± 0.0006 μm; *p*-value< 0.0001, *npm2a* and *npm2b*) to *npm2b* mutant follicles (0.0037 ± 0.0002 μm; *p*-value< 0.0001, *npm2a*) to *npm2a* mutant follicles (0.0025 ± 0.0002 μm). Only stage II follicles were quantitated, and the total areas of the follicles were comparable and not significantly different between all three groups (WT = 28,534 ± 2223 μm^2^, *npm2a* = 25,968 ± 1795 μm^2^, *npm2b* = 25,605 ± 2643 μm^2^) (Fig. 8c). Thus, our findings suggest that both *npm2a* and *npm2b* have roles in cortical granule function which may impact egg activation and fertilization.

The histological analyses of the ovaries of the mutant females demonstrated that there were significant structural perturbations in the *npm2a*- and *npm2b*-deficient follicles during early oogenesis that may negatively impact egg competence. Our analyses showed that there was a dramatic decrease in size and increase in number in CGs in stage II follicles of the *npm2a* mutants while there was a similar although not as striking difference in the *npm2b* mutants. Along with the results in Fig. 5 that showed very high levels of *npm2a* and *npm2b* expression from the earliest stage of oogenesis (stage I), our data suggest that these two genes may have important functions in the production and/or growth of CGs during early folliculogenesis. A previous study showed that the *souffle*/*spastizin* gene regulates secretory vesicle maturation such that a mutation in this gene resulted in accumulation of immature CGs and a defect in egg activation [49]. In addition, a mutation in the *aura* gene resulted in a decrease in CG exocytosis through its function in the reorganization of the cytoskeleton at fertilization [50]. Further, mutations in the *brom bones*/*hnrnpI* gene also led to loss of the Ca2+ signal induced by fertilization, subsequently, CG exocytosis was also decreased [51]. In these mutants, deficiency in CG maturity and exocytosis led to defects in egg activation, which was characterized by a decrease in cytoplasmic segregation and chorion membrane expansion, as well as in cell division, which exhibited as embryos with a cell mass atop an enlarged yolk syncytial region at the 1000-cell stage. Interestingly, we also observed dysfunctions in egg activation as the chorion membrane was not expanded fully in some of the eggs derived from *npm2a* mutant females (Fig. 7i, j, l), and the embryos from the *npm2b* females did demonstrate the same phenotype as the *aura* and *brom bones*/*hnrnpI* mutants corresponding to a defect in cell division (Fig. 7n and o). Thus, it seemed that the deformations in CGs in the *npm2a* stage II follicles led to a decrease in egg activation, and the *npm2b* mutants did not have a defect in egg activation, but in cell division.

Here, we demonstrated that while *npm2a* and *npm2b* share similar tissue distribution, as both are found specifically in the ovaries and early stage embryos, their roles are distinct and essential, and one could not compensate for the other. Notably, our data suggest that *npm2a* may play a large role in CG function, which regulates egg activation. In contrast, *npm2b* appears to play a minor role in CG function and to function at a later stage (ie. MBT) since the *npm2b*-deficient embryos are capable of dividing until around 4 hpf, which corresponds MBT as previously reported [5, 11], and mainly showed defects in cell division. These findings are in line with those demonstrated for other maternal-effect genes in mammals, such as *mater*, *floped*, *filia*, *tle6* [52], and *bcas2* [53] as well as in zebrafish, including *fue* [48] and *cellular atoll* [54], all of which function to moderate the early events of fertilization and embryogenesis and their ablation leads to arrest and mortality at the earliest stages of embryogenesis.

## Conclusions

Although the identification of maternal-effect genes and their functions is increasing, large gaps still remain as many maternal factors and regulatory mechanisms that contribute to the quality or developmental competence of the egg remain to be discovered. Our report describes clearly and in detail the evolutionary history, tissue distribution, as well as expression profile of the two *npm2* genes, and provides new findings that demonstrate their in vivo functions that clearly indicate that *npm2a* is a newly described maternal-effect gene. These results will help us gain further insight into the evolution of maternal-effect genes especially in a diverse range of vertebrates (chondrichthyes, sarcopterygians, and actinopterygians). The evolutionary history of the *npm2* genes indicated quite complex gene duplications and losses; an ancient gene duplication with subsequent loss of *npm2a* in mammals, and despite other WGD events, only one *npm2a* gene is retained in fish while *npm2b* was further duplicated during SaGD. It remains to be investigated whether the other copy of *npm2b* gene in salmonid is a pseudogene or not and if any other gene can functionally replace *npm2a* in mammals. Finally, our study will help us understand the underlying mechanisms that contribute to reproductive success, especially in terms of egg quality, in vertebrates.

## Additional files

Additional file 1: All the species used in each analysis. (XLSX 12 kb)

Additional file 2: List of Npm2 protein sequences used in the phylogenetic analysis. (XLSX 21 kb)

Additional file 3: qPCR and PCR primers. (XLSX 9 kb)

Additional file 4: Npm2a and Npm2b protein sequence comparisons. (XLSX 9 kb)

Additional file 5: List of the genes from the conserved syntenic region of *npm2* genes. (XLSX 16 kb)

Additional file 6: Npm2a and Npm2b protein domain conservation. Presence of the Npm core (red color bar), acidic tract A1 (green color bar), and acidic tract A2 (blue color bar) domains in *npm2a* and *npm2b* types in various vertebrate species. (EPS 77 kb)

## Abbreviations

CDS: Coding sequences
CGs: Cortical alveoli or granules
dN: Non-synonymous substitution rates
dS: Synonymous substitution rates
gRNA: Guide RNA
Mya: Million years ago
MZT: Maternal-to-zygotic transition
Npm2: Nucleoplasmin 2
ORF: Open reading frame
qPCR: Quantitative real-time PCR
RT: Reverse transcription
SaGD: Salmonid-specific genome duplication
SRA: Sequence Read Archive
TGD: Teleost-specific duplication
VGD: Vertebrate genome duplication
WGD: Whole genome duplication
WT: Wild-type
ZGA: Zygotic genome activation

## References

[1] Baroux C, Autran D, Gillmor CS, Grimanelli D, Grossniklaus U (2008). The maternal to zygotic transition in animals and plants. Cold Spring Harb Symp Quant Biol.

[2] Lindeman RE, Pelegri F (2010). Vertebrate maternal-effect genes: insights into fertilization, early cleavage divisions, and germ cell determinant localization from studies in the zebrafish. Mol Reprod Dev.

[3] Lee MT, Bonneau AR, Takacs CM (2013). Nanog, Pou5f1 and SoxB1 activate zygotic gene expression during the maternal-to-zygotic transition. Nature.

[4] Bouchareb A, Le Cam A, Montfort J (2017). Genome-wide identification of novel ovarian-predominant miRNAs: new insights from the medaka (Oryzias latipes). Sci Rep.

[5] Bouleau A, Desvignes T, Traverso JM (2014). Maternally inherited npm2 mRNA is crucial for egg developmental competence in zebrafish. Biol Reprod.

[6] Laskey RA, Honda BM, Mills AD, Finch JT (1978). Nucleosomes are assembled by an acidic protein which binds histones and transfers them to DNA. Nature.

[7] Laskey RA, Earnshaw WC (1980). Nucleosome assembly. Nature.

[8] Mills AD, Laskey RA, Black P, De Robertis EM (1980). An acidic protein which assembles nucleosomes in vitro is the most abundant protein in Xenopus oocyte nuclei. J Mol Biol.

[9] Philpott A, Leno GH, Laskey RA (1991). Sperm decondensation in Xenopus egg cytoplasm is mediated by nucleoplasmin. Cell.

[10] Philpott A, Leno GH (1992). Nucleoplasmin remodels sperm chromatin in Xenopus egg extracts. Cell.

[11] Burns KH, Viveiros MM, Ren Y (2003). Roles of NPM2 in chromatin and nucleolar organization in oocytes and embryos. Science (80-).

[12] Dehal P, Boore JL (2005). Two rounds of whole genome duplication in the ancestral vertebrate. PLoS Biol.

[13] Van de Peer Y, Maere S, Meyer A (2009). The evolutionary significance of ancient genome duplications. Nat Rev Genet.

[14] Eirin-Lopez JM, Frehlick LJ, Ausio J (2006). Long-term evolution and functional diversification in the members of the nucleophosmin/nucleoplasmin family of nuclear chaperones. Genetics.

[15] Frehlick LJ, Eirin-Lopez JM, Jeffery ED, Hunt DF, Ausio J (2006). The characterization of amphibian nucleoplasmins yields new insight into their role in sperm chromatin remodeling. BMC Genomics.

[16] Wotton KR, Weierud FK, Dietrich S, Lewis KE (2008). Comparative genomics of Lbx loci reveals conservation of identical Lbx ohnologs in bony vertebrates. BMC Evol Biol.

[17] Jovelin R, Yan YL, He X (2010). Evolution of developmental regulation in the vertebrate FgfD subfamily. J Exp Zool B Mol Dev Evol.

[18] Glasauer SM, Neuhauss SC (2014). Whole-genome duplication in teleost fishes and its evolutionary consequences. Mol Gen Genomics.

[19] Volff JN (2005). Genome evolution and biodiversity in teleost fish. Hered.

[20] Berthelot C, Brunet F, Chalopin D (2014). The rainbow trout genome provides novel insights into evolution after whole-genome duplication in vertebrates. Nat Commun.

[21] Pasquier J, Cabau C, Nguyen T (2016). Gene evolution and gene expression after whole genome duplication in fish: the PhyloFish database. BMC Genomics.

[22] Desvignes T., Fostier A., Fauvel C., Bobe J. (2012). The Nme gene family in fish. Fish Physiology and Biochemistry.

[23] Kimmel CB, Ballard WW, Kimmel SR, Ullmann B, Schilling TF (1995). Stages of embryonic development of the zebrafish. Dev Dyn.

[24] Selman K, Wallace RA, Sarka A, Qi X (1993). Stages of oocyte development in the zebrafish, Brachydanio rerio. J Morphol.

[25] Session AM, Uno Y, Kwon T (2016). Genome evolution in the allotetraploid frog Xenopus laevis. Nature.

[26] Near TJ, Eytan RI, Dornburg A (2012). Resolution of ray-finned fish phylogeny and timing of diversification. Proc Natl Acad Sci U S A.

[27] Tadros W, Lipshitz HD (2009). The maternal-to-zygotic transition: a play in two acts. Development.

[28] Laskey RA, Mills AD, Philpott A, Leno GH, Dilworth SM, Dingwall C (1993). The role of nucleoplasmin in chromatin assembly and disassembly. Philos Trans R Soc L B Biol Sci.

[29] Prado A, Ramos I, Frehlick LJ, Muga A, Ausio J (2004). Nucleoplasmin: a nuclear chaperone. Biochem Cell Biol.

[30] De La Fuente R, Viveiros MM, Burns KH, Adashi EY, Matzuk MM, Eppig JJ (2004). Major chromatin remodeling in the germinal vesicle (GV) of mammalian oocytes is dispensable for global transcriptional silencing but required for centromeric heterochromatin function. Dev Biol.

[31] Lee HH, Kim HS, Kang JY (2007). Crystal structure of human nucleophosmin-core reveals plasticity of the pentamer-pentamer interface. Proteins.

[32] Platonova O, Akey IV, Head JF, Akey CW (2011). Crystal structure and function of human nucleoplasmin (npm2): a histone chaperone in oocytes and embryos. Biochemistry.

[33] Okuwaki M, Sumi A, Hisaoka M (2012). Function of homo- and hetero-oligomers of human nucleoplasmin/nucleophosmin family proteins NPM1, NPM2 and NPM3 during sperm chromatin remodeling. Nucleic Acids Res.

[34] Lingenfelter BM, Tripurani SK, Tejomurtula J, Smith GW, Yao J (2011). Molecular cloning and expression of bovine nucleoplasmin 2 (NPM2): a maternal effect gene regulated by miR-181a. Reprod Biol Endocrinol.

[35] Namboodiri VM, Akey IV, Schmidt-Zachmann MS, Head JF, Akey CW (2004). The structure and function of Xenopus NO38-core, a histone chaperone in the nucleolus. Structure.

[36] Dutta S, Akey IV, Dingwall C (2001). The crystal structure of nucleoplasmin-core: implications for histone binding and nucleosome assembly. Mol Cell.

[37] Ramos I, Fernandez-Rivero N, Arranz R (2014). The intrinsically disordered distal face of nucleoplasmin recognizes distinct oligomerization states of histones. Nucleic Acids Res.

[38] Salvany L, Chiva M, Arnan C, Ausio J, Subirana JA, Saperas N (2004). Mutation of the small acidic tract A1 drastically reduces nucleoplasmin activity. FEBS Lett.

[39] Burglin TR, Mattaj IW, Newmeyer DD, Zeller R, De Robertis EM (1987). Cloning of nucleoplasmin from Xenopus laevis oocytes and analysis of its developmental expression. Genes Dev.

[40] Onikubo T, Nicklay JJ, Xing L (2015). Developmentally regulated post-translational modification of Nucleoplasmin controls histone sequestration and deposition. Cell Rep.

[41] Dingwall C, Dilworth SM, Black SJ, Kearsey SE, Cox LS, Laskey RA (1987). Nucleoplasmin cDNA sequence reveals polyglutamic acid tracts and a cluster of sequences homologous to putative nuclear localization signals. EMBO J.

[42] Banuelos S, Hierro A, Arizmendi JM, Montoya G, Prado A, Muga A (2003). Activation mechanism of the nuclear chaperone nucleoplasmin: role of the core domain. J Mol Biol.

[43] Taneva SG, Banuelos S, Falces J (2009). A mechanism for histone chaperoning activity of nucleoplasmin: thermodynamic and structural models. J Mol Biol.

[44] Fernandez-Rivero N, Franco A, Velazquez-Campoy A, Alonso E, Muga A, Prado A (2016). A quantitative characterization of Nucleoplasmin/histone complexes reveals chaperone versatility. Sci Rep.

[45] Harvey SA, Sealy I, Kettleborough R (2013). Identification of the zebrafish maternal and paternal transcriptomes. Development.

[46] Auer TO, Duroure K, De Cian A, Concordet JP, Del Bene F (2014). Highly efficient CRISPR/Cas9-mediated knock-in in zebrafish by homology-independent DNA repair. Genome Res.

[47] Gagnon JA, Valen E, Thyme SB (2014). Efficient mutagenesis by Cas9 protein-mediated oligonucleotide insertion and large-scale assessment of single-guide RNAs. PLoS One.

[48] Dekens MP, Pelegri FJ, Maischein HM, Nusslein-Volhard C (2003). The maternal-effect gene futile cycle is essential for pronuclear congression and mitotic spindle assembly in the zebrafish zygote. Development.

[49] Kanagaraj Palsamy, Gautier-Stein Amandine, Riedel Dietmar, Schomburg Christoph, Cerdà Joan, Vollack Nadine, Dosch Roland (2014). Souffle/Spastizin Controls Secretory Vesicle Maturation during Zebrafish Oogenesis. PLoS Genetics.

[50] Eno C, Solanki B, Pelegri F (2016). Aura/mid1ip1L regulates the cytoskeleton at the zebrafish egg-to-embryo transition. Development.

[51] Mei W, Lee KW, Marlow FL, Miller AL, Mullins MC (2009). hnRNP I is required to generate the Ca2+ signal that causes egg activation in zebrafish. Development.

[52] Li L, Baibakov B, Dean J (2008). A subcortical maternal complex essential for preimplantation mouse embryogenesis. Dev Cell.

[53] Xu Q, Wang F, Xiang Y (2015). Maternal BCAS2 protects genomic integrity in mouse early embryonic development. Development.

[54] Yabe T, Ge X, Pelegri F (2007). The zebrafish maternal-effect gene cellular atoll encodes the centriolar component sas-6 and defects in its paternal function promote whole genome duplication. Dev Biol.

